# Centenarians: who are they? A description of the total Swedish centenarian population in terms of living arrangements, health, and care utilization

**DOI:** 10.1007/s40520-023-02555-z

**Published:** 2023-09-05

**Authors:** Shunsuke Murata, Anna C. Meyer, Marcus Ebeling, Karin Modig

**Affiliations:** 1 Unit of Epidemiology, Institute of Environmental Medicine, Karolinska Institutet, Box 210, 17177 Stockholm, Sweden; 2 Department of Preventive Medicine and Epidemiology, National Cerebral and Cardiovascular Center, Osaka, Japan; 3 Laboratory of Population Health, Max Planck Institute for Demographic Research, Rostock, Germany

**Keywords:** Exceptional longevity, Long-term care, Home care services, Registries

## Abstract

**Background:**

The global centenarian population has doubled each decade and is expected to continue growing. However, information regarding how they live, their health status, and care needs is limited.

**Aims:**

This study aims to describe the total Swedish centenarian population in terms of health status, living arrangements, and socio-demographic characteristics.

**Methods:**

This nationwide register-based study included all Swedish people reaching age 100 between 2013 and 2018. We analyzed their socio-demographic characteristics, living arrangements, number of prescribed drugs, and health status. Moreover, their care transitions from age 100 and two years forward were described.

**Results:**

Of 5,882 centenarians (80.7% women), only 15.0% lived at home without formal care and 24.5% cohabited on their 100th birthday. Men (22.7%) were more likely than women (13.2%) to live at home without care. Approximately half of the centenarians lived in care homes, with fewer men (41.0%) than women (54.0%). Around 66.6% had a child living within the 50 km range. Most (76.5%) had an income below the median for Swedish older adults. Almost none were free from drugs, and polypharmacy was common (65.3%). Over half had at least one morbidity. Two years later, only 4.3% lived at home without care, and 63.9% died.

**Conclusion:**

Sweden’s centenarian population is highly dependent on home care and care homes. Among the ones still living at home, the vast majority live alone and have low incomes. Strategies to manage health and social care demands of this growing population group in the coming decade are important.

**Supplementary Information:**

The online version contains supplementary material available at 10.1007/s40520-023-02555-z.

## Introduction

The global number of centenarians—individuals who reach their 100th birthday—has roughly doubled every decade since 1950 and is projected to more than quintuple between 2022 and 2050 [1, 2]. It is often argued that centenarians are a selected and exceptionally healthy group. Indeed, centenarians have been shown to have fewer disabilities, hospitalizations, morbidities, better cognitive function in earlier life, and lower medical expenditures than their shorter-lived peers [3–8]. Still, most centenarians suffer from comorbidities and impairments in activities of daily living [3, 9]. Moreover, when more and more individuals survive their diseases and make it to 100, this population group might have changed in terms of health status and care needs and is perhaps frailer today than before. Despite the increasing interest in the oldest old, relatively little is known about this population.

Previous studies, based on survey data, Electric Health Records (EHRs), or sampled cohorts from various countries, have found different results regarding the health status of centenarians. The proportion of centenarians receiving home care or residing in care home has been reported to range from 26 to 54% [9–16]. Two Swedish studies analyzed polypharmacy, multimorbidity, and difficulties in activities of daily living (ADL) [3, 17] and found that polypharmacy was high and that almost all of the centenarians suffered from at least one morbidity and half of them from difficulties in ADL. Regarding living arrangements, studies from Portugal, Australia, USA, and Japan reported that less than 5% of centenarians lived alone and that over 75% had lost their spouse [13–16]. In Sweden, information on living arrangement was not reported.

However, only a few studies investigated centenarians’ characteristics in the entire population [10, 17]. Most previous studies were based on sampling data with some refusal rate or EHRs limited to individuals with specific medical insurance or individuals living in restricted areas, mostly urban areas [3, 11–14, 16, 17], which generally means a healthier population [18, 19]. In sampling studies, it is a critical concern that the consent to participate is related to sex, socio-economic status, and health status [20]. Selection effects may thus lead to an incomplete or biased picture of the centenarian population. Therefore, nationwide studies, including also institutionalized individuals, are required to provide the complete picture of the characteristics and care needs of this population. The Swedish population registers provide such an opportunity. This descriptive study thus aims to describe and understand the total centenarian population in Sweden, their socio-demographic characteristics, utilization of medical care, and geriatric care transitions.

## Methods

### Data source and study population

This study used a linkage of several nationwide population registers and included the total Swedish population that reached the age of 100 between February 2013 and December 2018. Baseline was set to February 2013 in order to identify the care status the month leading up to the 100th birthday for the inclusion of individuals in January 2013. The population was identified in the Total Population Register, to which the Social Service Register [21], the National Patient Register (NPR), the Prescribed Drug Register, the Dwelling Register, the Longitudinal Integrated Database for Health Insurance and Labour Market Studies (LISA) [22], the Multi-generation Register [23], and the National Cause of Death Register [24] were linked using the unique Swedish personal identification number.

When studying centenarians, it is crucial to verify their existence since some individuals might have emigrated from Sweden without officially reporting this and will then appear in the registers as a person without any hospital visits, geriatric care utilization, or death date. We performed a verification by checking whether individuals had any records in any of the three registers: the Social Service Register, the National Patient Register, and the Prescribed Drug register. If a person did not have any record for at least seven years before becoming a centenarian or after becoming a centenarian, we excluded them because we could not verify they were alive and residing in Sweden (n = 24). People were also excluded if they emigrated to other countries within the two-year follow-up period (n = 3), if they lived in a municipality not reporting consistently to the Social Service Register during the follow-up period (n = 89) [21], or had missing information on their socio-economic status in LISA (n = 242). The final study population consisted of 5,882 individuals. Given that the number of excluded individuals with loss-to-follow-up or missing values was small (5.4%), we believe that the population is representative for the total centenarian population in Sweden. To confirm this, we compared distribution differences in sex, degree of urbanization, the Charlson comorbidity index (CCI), and the number of drugs between individuals before and after excluding, which were very small (ranging from 0 to 0.7%, see Supplemental Table 1).

### Measurements and statistical analysis

We define geriatric care as the use of formal home care or residing in a care home and the information was extracted from the Social Service Register. We categorized participants into four groups: no formal care, receiving home care for less than 40 h per month, receiving home care for 40 h and more, and living in a care home. This was measured at baseline and after one year and two years, respectively. We calculated CCI using data from the NPR, in- and outpatient care, within ten years prior to the 100th birthday [25]. Information on drug use was extracted from the Prescribed Drug Register during six months prior to the 100th birthday. The number of prescribed drugs was counted based on the 3rd level of the Anatomical Therapeutic Chemical (ATC) code. Polypharmacy was defined as having five or more prescribed drugs of different kinds [26]. We further report the prevalence of the following specific drugs based on specific ATC codes: Anticoagulants (B01), Antihypertensive agents (C02), Diuretics (C03), Beta-blockers (C07), Calcium antagonists (C08), Agents affecting the renin-angiotensin system (RAS) (C09), Analgesics (N02), and Psychoanaleptics (N06), anti-dementia drugs (N06D).

Marital status, disposable income, and education were extracted from LISA. Disposable income was dichotomized based on the sex-and-year-specific median of people aged 80 years old or more and living alone [27]. To account for monetary inflation and sex differences, medians were calculated separately for each sex and year of income. Information on whether a person lived alone or cohabited was extracted from the Dwelling Register. Proximity to a child living in Sweden and in municipalities within 50 km was calculated using the Multi-generation Register [23] and the Total Population Register. The geographical distance between centenarians and their children was calculated based on the Euclidian distance between the centroids of municipalities where they live. Eurostat’s Degree of Urbanization (DEGURBA) classification system was used to determine the degree of urbanization. Two hundred ninety municipalities were classified into three groups: cities, towns and suburbs, and rural area [28, 29]. Information on socio-economic status was collected at the end of the year before reaching the 100th birthday.

Descriptive statistics were calculated for all included variables and presented as a total and sex and geriatric care stratified. Transitions of geriatric care between the 100th, 101st, and 102nd birthdays were illustrated using alluvial plots. All data were analyzed using R, version 4.1.2 [30].

## Results

### Socio-demographics

Table 1 shows the socio-demographic characteristics of the centenarians. Of the 5,882 individuals reaching the age of 100 years in Sweden between 2013 and 2018, 80.7% were women. Approximately half of the centenarians (51.5%) were residing in a care home when turning 100 years old, while the others lived at home with formal home care (33.5%) or with no formal home care (15.0%). Most centenarians had at least one child alive and living in Sweden (79.1%), and 2 out of 3 centenarians (66.6%) had at least one child within a geographical range of approximately 50 km. Their children’s mean age (SD, standard deviation) was 69.0 (5.7). The vast majority of centenarians (84.2%) were widowed. This proportion was higher among women than men, 86.9% versus 72.7%. Only 0.7% of the women were currently married compared to 18.8% of the men. The majority of the centenarians (66.0%) had only compulsory education, while 15.1% of the men and 7.8% of the women had undergraduate or graduate education. Most centenarians lived in urban (39.9%) or suburban areas (36.9%), and only 23.2% lived in rural areas. Men were more likely to live in rural areas than women (28.4% versus 22.0%). The majority (76.5%) had an income below the median of the 80+ population in Sweden. Among the centenarians, the median disposable income was higher in men 160,250 than in women 140,700.

**Table 1:** Demographic characteristics in centenarians

	Men (N = 1136)	Women (N = 4746)	Overall (N = 5882)
Living status, N (%)			
Live alone	468 (41.2%)	1906 (40.2%)	2374 (40.4%)
Cohabitation	202 (17.8%)	279 (5.9%)	481 (8.2%)
Care home	466 (41.0%)	2561 (54.0%)	3027 (51.5%)
Number of children, N (%)			
0	202 (17.8%)	1025 (21.6%)	1227 (20.9%)
1	276 (24.3%)	1330 (28.0%)	1606 (27.3%)
2 or more	658 (57.9%)	2391 (50.4%)	3049 (51.8%)
Number of children within 50 km, N (%)			
0	379 (33.4%)	1583 (33.4%)	1962 (33.4%)
1	368 (32.4%)	1724 (36.3%)	2092 (35.6%)
2 or more	389 (34.2%)	1439 (30.3%)	1828 (31.1%)
Marital status, N (%)			
Never married	56 (4.9%)	278 (5.9%)	334 (5.7%)
Married	214 (18.8%)	35 (0.7%)	249 (4.2%)
Divorced/separated	40 (3.5%)	308 (6.5%)	348 (5.9%)
Widower	826 (72.7%)	4125 (86.9%)	4951 (84.2%)
Education, N (%)			
Compulsory	653 (57.5%)	3231 (68.1%)	3884 (66.0%)
Upper secondary	311 (27.4%)	1144 (24.1%)	1,455 (24.7%)
Undergraduate or graduate level	172 (15.1%)	371 (7.8%)	543 (9.2%)
Urbanization, N (%)			
Cities	391 (34.4%)	1954 (41.2%)	2345 (39.9%)
Towns and suburbs	422 (37.1%)	1750 (36.9%)	2172 (36.9%)
Rural area	323 (28.4%)	1042 (22.0%)	1365 (23.2%)
Disposable income (SEK)			
Mean (SD)	229,617 (366,709)	176,175 (259,113)	186,496 (283,846)
Median [Q1, Q3]	160,250 (139,050, 210,200)	140,700 (124,900, 153,575)	142,800 (127,200, 161,400)
Disposable income < median^a^, N (%)	699 (61.5%)	3800 (80.1%)	4499 (76.5%)

*SD* standard deviation, *Q1* first quarter, *Q3* third quarter, *SEK* Swedish krona

^a^Disposable income was dichotomized based on the sex-year-specific median of people living alone and aged 80 or older

### Health status

**Fig. 1:**
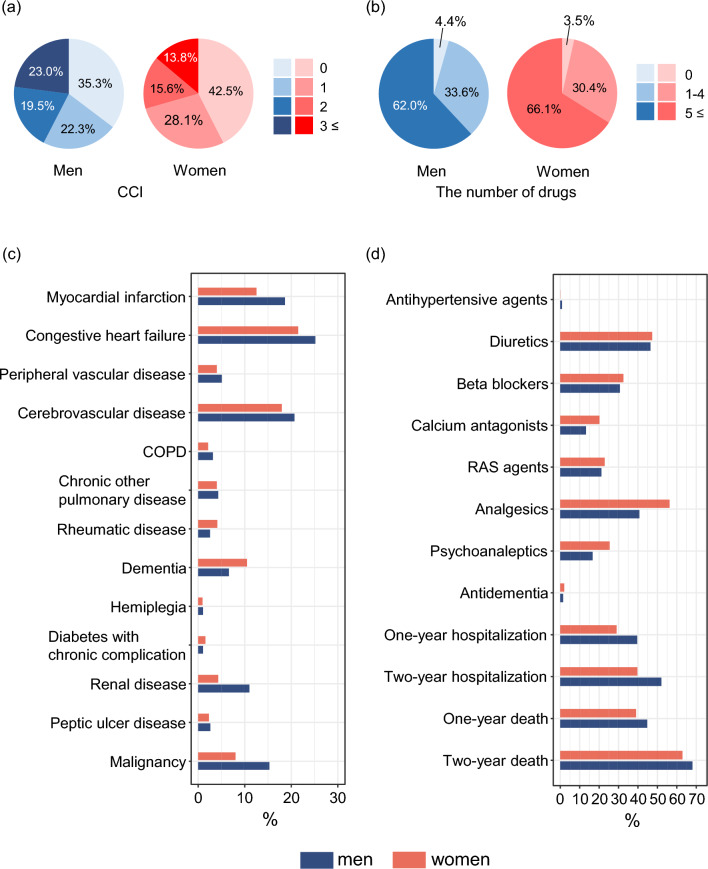
CCI, specific morbidity, drug information, hospitalizations, and death stratified by sex. Upper panel shows distribution of Charlson comorbidity index (**a**) and the number of drugs (**b**). Lower two panels show proportion of diseases (**c**) and drugs, hospitalizations, and death (**d**). *CCI* Charlson comorbidity index, *COPD* Chronic obstructive pulmonary disease, *RAS* Renin-angiotensin system

Figure 1 and Supplemental Table 2 present information regarding CCI, specific morbidity, drug prescriptions, hospitalizations, and death. The majority of centenarians suffered from at least one morbidity and 31.9% from multimorbidity. Men had more morbidities and higher mortality than women. The proportions of centenarians with a CCI score of 0 were 35.3 in men and 42.5 in women, and for CCI of 3 or more the proportions were 23.0% in men and 13.8% in women. Looking into the specific diagnoses that contribute to CCI revealed that heart failure (22.2%), followed by cerebrovascular disease (18.5%), were the most prevalent diagnoses among both men and women. The prevalence of dementia, rheumatic disease, and diabetes with chronic complications was higher in women than men, while that of other morbidities was higher in men than women.

The mean number (SD) of drugs among the centenarians was 6 (3). Very few, 3.7%, of the centenarians, had no prescribed drugs six months prior to their 100th birthday, while 65.3% had polypharmacy (i.e., five or more drugs). Women had more prescribed drugs than men. The proportion of polypharmacy was 66.1% in women and 62.0% in men. The most common drugs were analgesics which were prescribed to 53.3% of the centenarians. Prescriptions of diuretics and anticoagulants were also high with proportions of 47.2% and 45.5%. The proportion of centenarians prescribed anti-dementia drugs was 2.0%. For most drugs the proportions were higher in women than men except for anticoagulants (52.3% versus 43.8%) and antihypertensive agents (1.0% versus 0.1%) which were more common in men.

Supplemental Table 3 shows the two-year risk of hospitalization and death with stratification by sex and baseline geriatric care status. Men had a higher risk of hospitalization during two years of follow-up than women (52.1% in men versus 39.8% in women). In both men and women, centenarians receiving home care had the highest risk of becoming hospitalized, while centenarians living in care homes had the lowest risk. However, the risk of death was highest among centenarians living in care homes, followed by centenarians receiving home care and those without care.

**Fig. 2:**
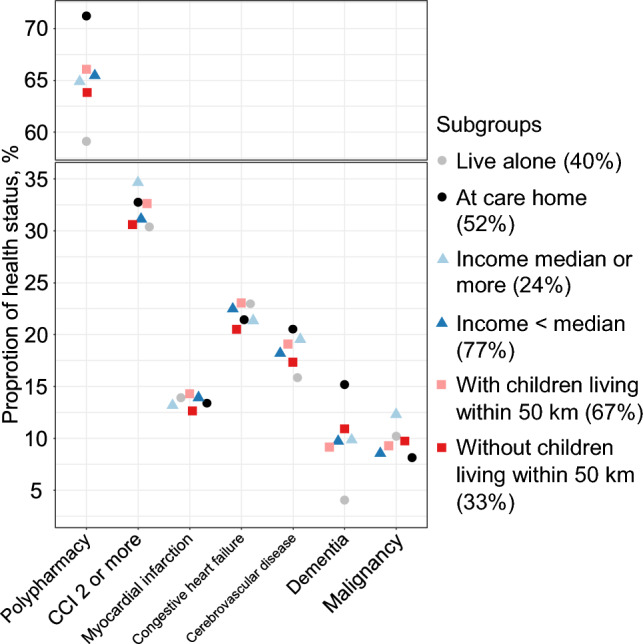
Proportion of polypharmacy, multimorbidity, and specific morbidity with stratification by living arrangement, income below median or not, and existence of children within 50 km. Y-axis shows the proportion with polypharmacy, CCI 2 or more, and prevalent disease. Point color reflects subgroup. Disposable income was dichotomized based on the sex-and-year-specific median of people living alone aged 80 years old or more. *CCI* Charlson comorbidity index

Figure 2 shows proportions of a number of health outcomes for three different stratifications of the centenarian population; those living at home or in care home, those with lower or higher income, and those with children living nearby or not. Overall, the health outcomes were similar between the subgroups. Only for polypharmacy and dementia there were a clear differences between centenarians living at home (59.1% polypharmacy and dementia 4.0%) and centenarians residing in care homes (polypharmacy, 71.2%; dementia, 15.2%).

### Geriatric care transitions

**Fig. 3:**
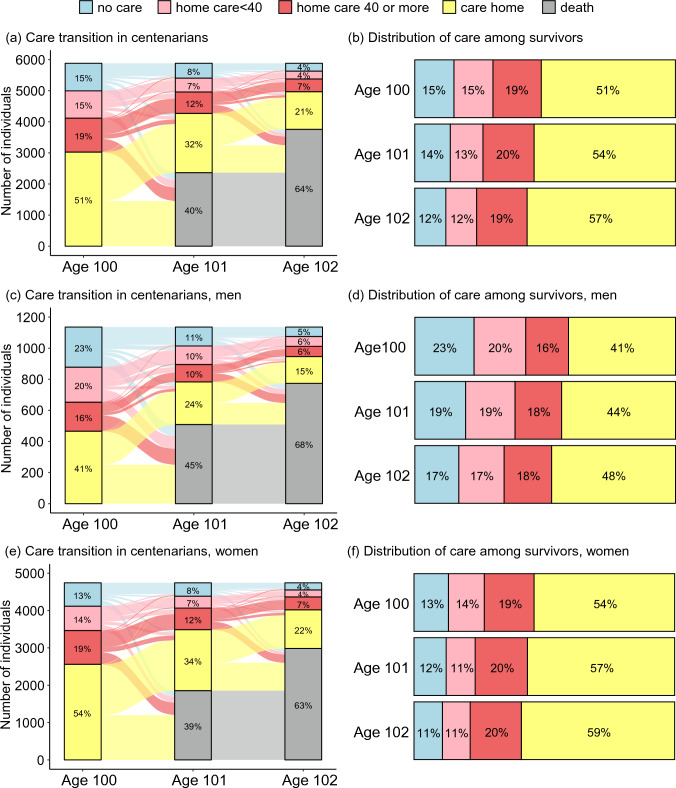
Two-year care transition in centenarians. Left column shows care and death transitions, and right column shows proportion of care status in survivors. Panel (**a**) and (**b**) are results of total centenarians. Panel (**c**) and (**d**) are results of men. Panel (**e**) and (**f**) are results of women

Figure 3 shows the two-year care transitions for men and women, respectively. Between age 100 and age 101, 40.2% of the centenarians had died; and after two years, 63.9% had died. Among the survivors, 56.9% resided in a care home, and 43.1% lived at home on their 102nd birthday. Among those living at home, 27.7% still had no formal home care. While the proportion of men living at home without care was higher than that of women throughout the follow-up, men were more likely to die. We additionally described care status restricting to subgroups living alone (Supplemental Table 4). The difference in the proportion receiving no care between men and women became narrower (baseline proportion of no care: men, 33.1%; women, 26.9%), while the proportion of death was still higher in men than in women (two-year risk of death: men, 61.5%; women, 52.0%).

## Discussion

In the present study, we have analyzed the total centenarian population in Sweden and shown that the vast majority of the centenarians were women, widowed, and lived in urban or suburban areas. Most centenarians who lived in the community lived alone, but most had children, many of whom lived close by. Four out of 10 centenarians had no comorbidities according to CCI. However, about two out of three centenarians had polypharmacy. Only 2% had no comorbidities, a maximum of 1 drug, and no geriatric care.

We observed that half of the centenarians lived in care homes. This proportion is very similar to what has been observed in studies from Denmark, Germany, Australia, and Canada [9, 11, 12, 15]. In contrast, some studies in Portugal, the US, and Japan reported lower proportions of centenarians residing in care homes, ranging from 26 to 32% [10, 14, 16]. These differences may be due to differences in informal care resources such as support through cohabiting partners, children, or other close kin. Sweden is a country where many older adults live alone [31], which is similar to Denmark, Germany, Australia, and Canada, while cohabitation with close kin seems be more common in Portugal, Japan, or the US [32, 33], perhaps reflecting higher shares of centenarians living in care homes in Sweden as compared to what has been reported in Portugal, Japan, or the US. We found that only 15% of the centenarians lived at home without formal home care at the age of 100. Two studies in Canada and Germany reported proportions of 20% and 6% in individuals aged 100 or older [11, 12]. However, the study from Germany also assessed home care provided by family members, which could explain why the proportion with no care is higher in Sweden than in the German study. Differences in the structure of health care systems might also explain parts of the variation in the proportions.

No study has presented transitions of geriatric care among centenarians. Our study reported that most centenarians died or increased their care needs during the two years after they turned 100, and that only 4% lived without any formal care after 2 years. Centenarians who utilized a large amount of care had a higher risk of death. Even if centenarians living at home were overall healthier than those residing in care homes, hospitalization was still more common in centenarians living at home. This may partly be explained by higher mortality in the care home group and by the fact that some medical conditions are being treated within the care homes.

Our work highlights some sex differences among Swedish centenarians. Men were more likely to cohabitate, used formal geriatric care to a lesser extent, and less often resided in care homes than women. Yet, men had more morbidities and higher mortality but fewer prescribed drugs than women. When restricting to centenarians living alone, the sex difference in the proportion of centenarians receiving no care became smaller, suggesting that care from a cohabiting partner account for part, but not all, of the lower care utilization among the men. This sex difference in geriatric care has also been shown in Canada, Germany, and France [11, 12, 34]. Also, the sex-specific results on disease prevalence and drug prescription resemble those of earlier studies from Canada and Spain [11, 35].

We observed that the majority of centenarians had polypharmacy, in line with what has been reported previously in Sweden and Spain [17, 35]. The high drug use is in line with the high proportion of multimorbidity among centenarians, also observed in previous studies [3, 9, 12, 35]. Yet, given the high mortality and the high age it may be questioned if all those drugs are motivated.

Overall, we found similar health status across subgroups of the centenarian population. Thus, low socio-economic status seems not to matter too much in terms of the health status among centenarians. However, it is essential to note that the majority live alone and have a low income, making most of them belong to a vulnerable group with a potential lack of social support. A clear difference was observed in terms of dementia prevalence so that individuals living in care homes more often had polypharmacy and dementia than those living alone. While dementia may prompt the move to a care home, it is unclear if polypharmacy increases the risk of entering a care home, or if living in a care home leads to more polypharmacy. Further studies to explore pathways of these factors are necessary.

### Strength and limitations

The main strength of this paper is that it includes all centenarians living in Sweden, without exclusion of those living in care homes or those unable to respond due to poor health. The health information is virtually complete for hospitalizations, drug prescriptions, and formal geriatric care. However, there are also limitations. First, we were unable to assess the provision of informal or privately paid home care. As such, the observed care status may not perfectly reflect an individual’s care needs. Still, publicly funded care is heavily subsidized in Sweden and available to every Swedish resident. Only a few centenarians in our study received no publicly funded care, which may indicate that those in need most often draw on publicly-funded resources. Second, morbidities were defined according to the CCI, one of the most commonly used morbidity indices in epidemiological and clinical research. Still, it only captures a specific selection of diseases. We were not able to assess other dimensions of health including cognitive and physical functioning, self-rated health, or pain. The prevalence of dementia was measured by a diagnosis code in the National Patient Register. This has been shown to have high positive predictive value but lower sensitivity [36], resulting in an underestimation of the true prevalence.

## Conclusions

Centenarians are sometimes described as a health-selected group of individuals who have escaped age-related diseases. However, in this nationwide descriptive study, we show that Swedish centenarians are not very healthy or independent, but suffer from comorbidities, polypharmacy, and are largely dependent on geriatric care. They are also vulnerable in terms of socio-demographics and economic resources since most live alone and have a low income. Therefore, strategies to manage health and social care demands of this growing population group are important.

### Supplementary Information

Below is the link to the electronic supplementary material.

Supplementary file1 (PDF 116 KB)

## Data Availability

All data supporting the findings of this study are available within the paper and its Supplementary Information.
