# Supplementary material for: Centenarians: who are they? A description of the total Swedish centenarian population in terms of living arrangements, health, and care utilization
Source: Aging Clin Exp Res. 2023 Sep 5;35(11):2759–67. doi: 10.1007/s40520-023-02555-z (PMC10628024; doi:10.1007/s40520-023-02555-z)
Supplement: Supplementary file 1 — Supplementary file1 (PDF 116 KB) [file 40520_2023_2555_MOESM1_ESM.pdf]

Centenarians: who are they? A description of the total Swedish centenarian population in terms of living arrangements, health and care utilization.

Shunsuke Murata[1,2], Anna C Meyer[1], Marcus Ebeling[1, 3], Karin Modig[1]

1. Unit of Epidemiology, Institute of Environmental Medicine, Karolinska Institutet, Stockholm, Sweden
2. Department of Preventive Medicine and Epidemiology, National Cerebral and Cardiovascular Center, Osaka, Japan
3. Laboratory of Population Health, Max Planck Institute for Demographic Research, Rostock, Germany

Correspondence: Shunsuke Murata, Unit of Epidemiology, Institute of Environmental Medicine, Karolinska Institutet, Box 210, 17177 Stockholm, Sweden

Email: shunsuke.murata@ki.se

Supplementary Table 1. Comparison of sex, Charlson comorbidity index, and the number of drugs between analytic population and population before excluding.

	Analytic population (N=5882)	Population before excluding (N=6216)
Sex, N (%)		
men	1,136 (19.3%)	1,205 (19.4%)
women	4,746 (80.7%)	5,011 (80.6%)
Urbanization, N (%)		
Cities	2,345 (39.9%)	2,518 (40.5%)
Towns and suburbs	2,172 (36.9%)	2,277 (36.6%)
Rural area	1,365 (23.2%)	1,421 (22.9%)
Charlson comorbidity index, N (%)		
0	2,417 (41.1%)	2,559 (41.2%)
1	1,586 (27.0%)	1,672 (26.9%)
2	961 (16.3%)	1,021 (16.4%)
3 or more	918 (15.6%)	964 (15.5%)
Number of drugs, N (%)		
0	215 (3.7%)	238 (3.8%)
1–4	1,826 (31.0%)	1,920 (30.9%)
5–	3,841 (65.3%)	4,058 (65.3%)

Supplementary Table 2. Charlson comorbidity index, specific morbidity, drug information, hospitalizations, and death in centenarians

	Men (N=1,136)	Women (N=4,746)	Overall (N=5,882)
Charlson comorbidity index, N (%)			
0	401 (35.3%)	2,016 (42.5%)	2,417 (41.1%)
1	253 (22.3%)	1,333 (28.1%)	1,586 (27.0%)
2	221 (19.5%)	740 (15.6%)	961 (16.3%)
3 or more	261 (23.0%)	657 (13.8%)	918 (15.6%)
Specific morbidity, N (%)			
Congestive heart failure	286 (25.2%)	1,019 (21.5%)	1,305 (22.2%)
Cerebrovascular disease	235 (20.7%)	853 (18.0%)	1,088 (18.5%)
Myocardial infarction	212 (18.7%)	596 (12.6%)	808 (13.7%)
Dementia	75 (6.6%)	498 (10.5%)	573 (9.7%)
Malignancy	174 (15.3%)	380 (8.0%)	554 (9.4%)
Renal disease	125 (11.0%)	204 (4.3%)	329 (5.6%)
Peripheral vascular disease	58 (5.1%)	189 (4.0%)	247 (4.2%)
Chronic other pulmonary disease	49 (4.3%)	190 (4.0%)	239 (4.1%)
Rheumatic disease	29 (2.6%)	195 (4.1%)	224 (3.8%)
Chronic obstructive pulmonary disease	36 (3.2%)	102 (2.1%)	138 (2.3%)
Peptic ulcer disease	30 (2.6%)	109 (2.3%)	139 (2.4%)
Diabetes with chronic complication	12 (1.1%)	74 (1.6%)	86 (1.5%)
Hemiplegia	12 (1.1%)	45 (0.9%)	57 (1.0%)
Number of drugs			
Mean (SD)	5.7 (3.2)	6.0 (3.3)	6.0 (3.3)
Median [Q1, Q3]	5 (3, 8)	6 (4, 8)	6 (4, 8)
The drugs number, N (%)			
0	50 (4.4%)	165 (3.5%)	215 (3.7%)
1–4	382 (33.6%)	1,444 (30.4%)	1,826 (31.0%)
5 or more (polypharmacy)	704 (62.0%)	3,137 (66.1%)	3,841 (65.3%)
Specific drugs, N (%)			
Analgesics	463 (40.8%)	2,672 (56.3%)	3,135 (53.3%)
Diuretics	528 (46.5%)	2,246 (47.3%)	2,774 (47.2%)
Anticoagulants	594 (52.3%)	2,081 (43.8%)	2,675 (45.5%)
Beta blockers	349 (30.7%)	1,544 (32.5%)	1,893 (32.2%)
Psych analeptics	190 (16.7%)	1,211 (25.5%)	1,401 (23.8%)
Agents affecting renin angiotensin system	241 (21.2%)	1,089 (22.9%)	1,330 (22.6%)
Calcium antagonists	151 (13.3%)	959 (20.2%)	1,110 (18.9%)
Antidementia	17 (1.5%)	98 (2.1%)	115 (2.0%)
Antihypertensive agents	11 (1.0%)	5 (0.1%)	16 (0.3%)
One-year hospitalization, N (%)	450 (39.6%)	1,381 (29.1%)	1,831 (31.1%)
Two-year hospitalization, N (%)	592 (52.1%)	1,887 (39.8%)	2,479 (42.1%)
One-year death, N (%)	509 (44.8%)	1,854 (39.1%)	2,363 (40.2%)
Two-year death, N (%)	774 (68.1%)	2,986 (62.9%)	3,760 (63.9%)

SD, standard deviation; Q1, first quarter; Q3, third quarter

Supplementary Table 3. Incidence proportion of hydration and death stratified by sex and baseline care status.

	Men			Women		
	no care (N = 258)	home care (N = 412)	care home (N = 466)	no care (N = 627)	home care (N = 1,558)	care home (N = 2,561)
One-year hospitalization, N (%)	117 (45.3%)	225 (54.6%)	108 (23.2%)	248 (39.6%)	725 (46.5%)	408 (15.9%)
Two-year hospitalization, N (%)	159 (61.6%)	283 (68.7%)	150 (32.2%)	356 (56.8%)	968 (62.1%)	563 (22.0%)
One-year death, N (%)	76 (29.5%)	183 (44.4%)	250 (53.6%)	169 (27.0%)	485 (31.1%)	1,200 (46.9%)
Two-year death, N (%)	137 (53.1%)	278 (67.5%)	359 (77.0%)	287 (45.8%)	865 (55.5%)	1,834 (71.6%)

Supplementary Table 4. Care status and hospitalizations in centenarians living alone at home.

	Men (N=468)	Women (N=1,906)	Overall (N=2,374)
Care status, N (%)			
no care	155 (33.1%)	513 (26.9%)	668 (28.1%)
home care<40	163 (34.8%)	578 (30.3%)	741 (31.2%)
home care over 40	150 (32.1%)	815 (42.8%)	965 (40.6%)
Care status after 1 year, N (%)			
no care	76 (16.2%)	288 (15.1%)	364 (15.3%)
home care<40	91 (19.4%)	294 (15.4%)	385 (16.2%)
home care over 40	86 (18.4%)	518 (27.2%)	604 (25.4%)
care home	39 (8.3%)	247 (13.0%)	286 (12.0%)
death	176 (37.6%)	559 (29.3%)	735 (31.0%)
Care status after 2-year, N (%)			
no care	37 (7.9%)	154 (8.1%)	191 (8.0%)
home care<40	46 (9.8%)	172 (9.0%)	218 (9.2%)
home care over 40	51 (10.9%)	311 (16.3%)	362 (15.2%)
care home	46 (9.8%)	277 (14.5%)	323 (13.6%)
death	288 (61.5%)	992 (52.0%)	1,280 (53.9%)
One-year hospitalization, N (%)	237 (50.6%)	846 (44.4%)	1,083 (45.6%)
Two-year hospitalization, N (%)	312 (66.7%)	1,149 (60.3%)	1,461 (61.5%)
